# Qualitative and Quantitative Diagnosis in Head and Neck Cancer

**DOI:** 10.3390/diagnostics11091526

**Published:** 2021-08-24

**Authors:** Fernando López, Antti Mäkitie, Remco de Bree, Alessandro Franchi, Pim de Graaf, Juan C. Hernández-Prera, Primoz Strojan, Nina Zidar, Margareta Strojan Fležar, Juan P. Rodrigo, Alessandra Rinaldo, Barbara A. Centeno, Alfio Ferlito

**Affiliations:** 1 Department of Otorhinolaryngology, Head and Neck Surgery, Hospital Universitario Central de Asturias, 33011 Oviedo, Spain; jprodrigo@uniovi.es; 2 Instituto de Investigación Sanitaria del Principado de Asturias (ISPA), Instituto Universitario de Oncología del Principado de Asturias (IUOPA), University of Oviedo CIBERONC-ISCIII, 33011 Oviedo, Spain; 3 Department of Otorhinolaryngology–Head and Neck Surgery, University of Helsinki and Helsinki University Hospital, 00029 Helsinki, Finland; Antti.Makitie@hus.fi; 4 Department of Head and Neck Surgical Oncology, University Medical Center Utrecht, 3584CX Utrecht, The Netherlands; R.deBree@umcutrecht.nl; 5 Department of Translational Research, School of Medicine, University of Pisa, 56124 Pisa, Italy; alessandro.franchi@unipi.it; 6 Cancer Center Amsterdam, Department of Radiology and Nuclear Medicine, Amsterdam UMC, Vrije Universiteit Amsterdam, 1081 Amsterdam, The Netherlands; p.degraaf@amsterdamumc.nl; 7 Department of Pathology, Moffitt Cancer Center, Tampa, FL 33612, USA; Juan.Hernandez-Prera@moffitt.org (J.C.H.-P.); Barbara.Centeno@moffitt.org (B.A.C.); 8 Department of Radiation Oncology, Institute of Oncology, 1000 Ljubljana, Slovenia; pstrojan@onko-i.si; 9 Department of Head and Neck Pathology, Faculty of Medicine, Institute of Pathology, University of Ljubljana, 1000 Ljubljana, Slovenia; nina.zidar@mf.uni-lj.si; 10 Department of Cytopathology, Faculty of Medicine, Institute of Pathology, University of Ljubljana, 1000 Ljubljana, Slovenia; margareta.strojan-flezar@mf.uni-lj.si; 11 University of Udine School of Medicine, 33100 Udine, Italy; alessandra.rinaldo@uniud.it; 12 Coordinator of the International Head and Neck Scientific Group, 35100 Padua, Italy; profalfioferlito@gmail.com

**Keywords:** quantitative diagnosis, qualitative diagnosis, head and neck

## Abstract

The diagnosis is the art of determining the nature of a disease, and an accurate diagnosis is the true cornerstone on which rational treatment should be built. Within the workflow in the management of head and neck tumours, there are different types of diagnosis. The purpose of this work is to point out the differences and the aims of the different types of diagnoses and to highlight their importance in the management of patients with head and neck tumours. Qualitative diagnosis is performed by a pathologist and is essential in determining the management and can provide guidance on prognosis. The evolution of immunohistochemistry and molecular biology techniques has made it possible to obtain more precise diagnoses and to identify prognostic markers and precision factors. Quantitative diagnosis is made by the radiologist and consists of identifying a mass lesion and the estimation of the tumour volume and extent using imaging techniques, such as CT, MRI, and PET. The distinction between the two types of diagnosis is clear, as the methodology is different. The accurate establishment of both diagnoses plays an essential role in treatment planning. Getting the right diagnosis is a key aspect of health care, and it provides an explanation of a patient’s health problem and informs subsequent decision. Deep learning and radiomics approaches hold promise for improving diagnosis.

## 1. Introduction

A feature of head and neck cancer (HNC) is that both the disease and treatment carry frequent acute and late toxicities that can have profound effects on patients’ quality of life (QoL) because it can affect essential functions, such as swallowing, speech, and breathing [1,2]. Treatment of HNC may consist of surgery followed by radiotherapy (RT) with or without chemotherapy (CT) or RT, with or without CT, with salvage surgery in reserve [3]. Each treatment modality harbours its own specific morbidity. Therefore, when choosing the most appropriate treatment, it is essential to have an accurate diagnosis to avoid mismanagement of the patient with impact on complications, QoL, and survival.

The diagnostic workup is challenging because of the heterogeneity and complexity of HNC [4,5]. Diagnosis remains the art of determining the nature and extent of a disease condition [6]. Clinical decision making in head and neck oncology aims to offer the best management options for the patient. Based on the patient’s medical history, performance and nutritional status, comorbidities, physical symptoms, and signs, and, often, on the results of laboratory tests, radiological imaging, and pathological and molecular investigations [6], the multidisciplinary tumour board (MTB) will thus apply all relevant information that is related either to the patient or the tumour and available treatment modalities to offer the best management options for the patient.

According to the actions performed and the tools used to carry out a diagnosis, there are several types of diagnosis as described by Ferlito et al. [7]. However, there are two types that will be essential when determining the best management of patients: qualitative and quantitative diagnosis.

The most important qualitative diagnosis is the conventional pathological diagnosis, which is provided by a pathologist sometimes assisted by subspecialists in pathology, such as cytopathologist or molecular biologists. The choice of the correct treatment and the patient’s prognosis often ultimately depends on a correct pathological diagnosis based on morphological evaluation of tissue or cell samples of the tumour, which also use adjunct studies. On the other hand, examples of quantitative diagnosis are the identification of a mass lesion, the estimation of the tumour volume, its composition, and its local, regional, and distant extent. It is mainly provided by the radiologist and nuclear medicine specialist. Quantitative diagnosis is part of the clinical diagnosis together with the patient’s medical history and physical examination. However, all this must be corroborated by pathological diagnosis.

The purpose of this work is to point out the differences between both types of diagnoses and to highlight the importance of each in the management of patients with HNC.

## 2. Qualitative Diagnosis

An accurate diagnosis of the type of malignancy is a key component of effective management [8]. This diagnosis should be made by expert pathologists who should be a core member of the cancer MTB because they are essential to the provision of a successful service. It is particularly important to make accurate diagnoses and reduce diagnostic uncertainty to avoid misunderstandings and mistakes between clinicians when formulating a treatment plan [9]. Although the initial diagnosis may be obvious clinically based on an irregularly infiltrating mass with or without ulceration, it should always be confirmed by biopsy. Some diseases, e.g., tuberculosis and sarcoidosis, can mimic tumours clinically, and some malignancies, e.g., lymphoma, may require consideration of other treatment options.

Pathologists play critically important roles in confirming or excluding specific diseases based on cell sample or tissue biopsy, in recognizing key predictive and prognostic factors, assessing the adequacy of treatment, and in contributing evidence-based criteria for the appropriate stratification of clinical outcomes.

The pathologist is also responsible for providing many of the more specific data elements that will guide treatment decisions: examples include evidence of virally driven malignancy, margin status, and the precise depth to which a tumour invades surrounding normal tissue (in the latter case, although it is a quantitative diagnosis, it is established within the pathology report).

To enhance communication among clinicians and ultimately to improve patients’ care and clinical outcomes, pathology reports containing all the required information for treatment should be standardized. For example, data sets for reporting head and neck malignancies have been developed based on current evidence by the International Collaboration for Cancer Reporting (ICCR) [10]. These include all the anatomic sites plus mucosal malignant melanoma and nodal excision and neck dissections. These datasets define the essential pathological data that are required for diagnosis, staging, and assessment of prognosis of head and neck cancer patients. The final aim is to ensure that all the relevant information determined on current knowledge is included in the pathology report and presented in a consistent format in agreement with international standards.

Moreover, it is important to note that there are some aspects of the pathological diagnosis, or at least of the data that are part of a pathology report, that can be considered quantitative. Depth of invasion (DOI) measurement in oral tongue carcinomas [11], the mitotic index [12] and the quantification of immunostaining (Ki67 labelling index [12], p16 positivity [13] as surrogate marker for HPV infection, or programmed death-ligand 1 (PD-L1) as immunotherapeutic target [14] are some of the measurements that should be routinely included in pathological report.

The first step in diagnosis is the microscopic morphologic analysis of a sample of tumour cells or tissue. An in-office biopsy with forceps (incisional biopsy of the primary tumour) and/or fine-needle aspiration (FNA) cytology (in suspicious lymph nodes) are usually the initial sources of diagnostic samples. Excisional biopsy of a neck mass is not recommended unless the FNA biopsy sample has been persistently non-diagnostic, a primary site has not been identified on cross-sectional imaging or pan-endoscopy, and/or lymphoma is suspected owing to concurrent non-cervical lymphadenopathy. In these small cell and tissue samples, there is sometimes limited material for extensive pathological studies, so larger tissue biopsies, at least core needle biopsy, or samples from surgical resection are necessary to make an accurate diagnosis. However, as a rule, surgical resection follows a previously made histopathological diagnosis.

Tissue specimens should be submitted in an appropriate quantity and placed immediately after removal in a fixative solution, 10% neutral formalin being the most used in pathology labs. A discussion of the pros and cons of each fixative is beyond the scope of the paper. The site and nature of each sample should be clearly described on the application form and appropriately oriented. The request for analysis form should include clinical data to guide the pathologist. Besides, proper handling of cell samples obtained either by free-hand or ultrasound-guided FNAC is also mandatory for reliable diagnosis of the tumour.

Intra-operative frozen sections are a useful tool if appropriately used, especially for the assessment of surgical excision margins when there is clinical doubt as to their possible involvement [15]. It should be noted that the quality of frozen sections is not as good as that of paraffin sections and that critical information may be lost, especially if disoriented and small pieces of tissue are submitted for examination or in the case of tumours with complex histopathological pattern. In addition, frozen section diagnosis requires pathologists with experience of integrating surgical and pathology findings and the capacity to make quick and accurate decisions.

The basic pathological study consists of a haematoxylin-eosin (HE) stain. By means of this staining, it is possible to make a diagnostic orientation of a large majority of tumours and to evaluate their degree of differentiation [16]. Histological grading is often of limited value due to its lack of prognostic impact [17]. However, in addition to histologic grading, there are other histopathologic parameters (e.g., pattern of invasive front, tumour budding and cell nest size, perineural invasion and lymphovascular invasion) that must be assessed using HE staining and can aid in understanding the behaviour of each individual case. Lymphatic and vascular invasion are associated with a high probability of lymph node and distant metastases, respectively [18]. Similarly, perineural invasion is associated with an increased risk of local recurrence, regional lymph node metastases, and poorer survival [19]. Another important feature is the presence of extra-nodal extension of metastases. It is strongly associated with both regional recurrence and the development of distant metastases, resulting in poorer survival. It has therefore been introduced as a descriptor in the 8^th^ edition of AJCC (UICC) staging for HPV-negative squamous cell carcinoma of the larynx. Growth pattern at the invasive tumour front is another important feature with prognostic implication: an infiltrative pattern with extensive tumour budding is associated with a more aggressive course and poorer prognosis than an expansive pattern. The pattern of invasion has proven to have prognostic value in oral squamous cell carcinoma (SCC) and can be distinguished as cohesive, non-cohesive, or dispersed [20]. The presence of tumour satellites 1 mm or more from the main tumour or nearest satellite is a recognized adverse prognostic factor, especially in SCC of the tongue [20].

Moreover, HE slides can be used to report various other parameters that add to the diagnosis and have been suggested to be included in tumour classification: tumour depth, tumour budding, tumour-infiltrating lymphocytes (TILs), or cell-in-cell phenomenon [21]. In FNA samples, Giemsa and Papanicolaou staining are usually used after appropriate on-site fixation of FNA cell samples. Rapid on-site evaluation (ROSE) of the cell sample can be used to assure representative and satisfactory sample for cytomorphological evaluation and adjunct studies. In FNA cell samples, orange cytoplasm of malignant cells in Papanicolaou suggests keratinization and squamous differentiation.

Practical problems that can hinder a correct diagnosis in biopsies are poor orientation, sampling errors, morphological overlap between different entities, the presence of necrotic debris or too much inflammatory reaction, small samples with few cells, and crush artefacts. Laser-resection specimens can also be difficult to diagnose because they often have thermal artefacts, making detailed interpretation impossible. Patients who have been treated with RT and/or CT may have extensive scar tissue, radiation-associated nuclear atypia, and loss of normal anatomical landmarks, which can make evaluation of these specimens difficult. A good response to CT may leave a mass of necrotic tissue that prevents the observation of viable tumour even after extensive histological sampling.

### 2.1. Immunohistochemistry

Immunohistochemistry (IHC) plays an important role in the correct diagnosis of primary HNC, particularly for the less common entities. Currently, digital methods to improve analysis and diagnostic data are available [22]. Their main goals are to standardize diagnostic procedures and to provide reliable and reproducible results. Although the determination and quantification of automated immunohistochemical staining allows to obtain quality results with high reproducibility, it is important to bear in mind that the information of an experienced histopathologist is essential in complex cases and to avoid errors in case the diagnostic algorithms are poorly calibrated.

There are currently immunohistochemical markers that are routinely performed to confirm a diagnosis, such as anti-Epithelial Membrane Antigen (EMA), p63, p40, or cytokeratins used in SCC. Moreover, IHC allows accurate classification of poorly differentiated or undifferentiated tumours and carcinomas of unknown primary site. In addition, IHC can solve diagnostic dilemmas in the differentiation of various tumours. Advances in IHC staining and genetic markers have improved diagnostic accuracy in salivary and neuroendocrine malignancies [23,24,25]. In both groups of neoplasms, there are different histological types with sometimes non-specific morphological characteristics. The distinction is critical given the impact on the therapeutic approach.

Some markers have emerged as being fundamental in the diagnosis of tumours, as they have prognostic and therapeutic implications. The prognostic value of assessing oropharyngeal carcinomas for evidence of human papilloma virus infection (HPV) is well established, with current guidance recommending routine IHC for p16 protein overexpression and in-situ hybridization for high-risk HPV DNA [13]. Moreover, morphologically similar poorly differentiated carcinomas arising in the oropharynx and nasopharynx and their nodal metastases may be distinguished by the presence of HPV and Epstein–Barr virus (EBV) DNA, respectively. The detection of the presence of these viruses is also important in the management of lymph node metastases of an unknown origin, as it can provide guidance to the location of the primary tumour. During the last years, PD-L1 testing by IHC has become standard of care in the management of recurrent and/or metastatic head and neck squamous cell carcinoma, and combined proportion score (CPS) ≥ 1 determines eligibility for treatment with pembrolizumab [26]. In addition, succinate dehydrogenase complex subunit B (SDHB) IHC stain should be routinely performed in all head and neck paragangliomas, and the loss of expression of this marker can help identifying patients with underlying genetic predisposition syndromes to develop these tumours [27].

Several well-known prognostic factors can be easily assessed by IHC, including the presence of mutations of the TP53 tumour-suppressor gene and the cell proliferation marker Ki-67. Other markers have emerged as potential prognostic factors, although their use has not yet entered the routine practice. NANOG and SOX2 protein expression is frequent in SCC; combined expression of both proteins showed the highest survival rates and double-negative cases the worst survival [28]. Moreover, markers such as cortactin (CTTN) and the focal adhesion kinase (FAK) and NANOG can be used as complementary markers for risk stratification in patients with laryngeal precancerous lesions [29,30,31,32,33]. IHC can also identify micrometastases and isolated tumour cells in lymph nodes [34], bone marrow [35], or in other distant sites [6] without finding atypical cells. Identification of more sensitive and specific new markers increases diagnostic accuracy while saving tissue and money. For example, it was found that insulinoma-associated protein 1 (INSM1) can successfully replace a panel of traditional markers (synaptophysin, chromogranin, and CD56) used for documentation of neuroendocrine differentiation [36].

In patients with lymph node metastases of unknown origin, the morphological features of the metastatic tumour may be a useful aid in finding its origin, e.g., thyroid and salivary neoplasms. Clinicians should be aware that immunohistochemical markers are often not entirely specific and that decisions about primary sites are based on the evaluation of a panel of different markers and on the balance of probabilities. Clinical features and imaging studies should be added into the multidisciplinary assessment of these patients. In general, molecular profiling is generally not currently recommended outside the research setting [37,38]. IHC of cytology samples, core biopsy samples, or tissue obtained by open biopsy as well as molecular studies, in some cases, can render a more accurate diagnosis. IHC may be helpful in identifying primary tumours of the oropharynx (p16+/HPV+) or the nasopharynx (EVB+), but it does not reliably distinguish between other primary sites of SCCs. The most challenging diagnostic issues occur in cases of adenocarcinoma because of the multiplicity of anatomic sites that may harbour such a primary tumour. A differential diagnosis for adenocarcinoma in neck lymph nodes includes metastases from the breast, lungs, gastrointestinal tracts, and prostate [39]. In such cases, primary salivary gland malignancies should also be considered in the differential diagnosis. Different tumour markers may aid in the identification of the site of the cancer. It is beyond the scope of this paper to list all available markers. Previous work of the IHNSG group has developed this topic extensively [40].

### 2.2. Molecular Analysis

Molecular analysis has been incorporated in current clinical practice with reference to the ability to specifically diagnose various tumours. Some tumours, such as small round cell tumours, are condensed into a single group by cytological and immunohistochemical analysis; however, the use of molecular biology techniques has made it possible to identify and isolate groups with a specific prognosis and treatment. These investigations have had an enormous impact in neuroendocrine tumours, sarcomas, and haematolymphoid tumours [25]. In addition, some tumours can now be identified by molecular genetic analysis, for example, NUT (NUclear protein in testis) carcinoma and SWI/SNF chromatin remodelling complex, particularly its subunits SMARCA4 (BRG1)- and SMARCB1 (INI-1)-deficient carcinomas [41,42]. An increasing number of salivary gland tumours have recurrent genetic alterations, mainly translocations and less frequently point mutations, that can be used to improve diagnostic accuracy and may serve as targets for new treatments [43].

Next-generation sequencing (NGS) allows the detection of genetic alterations, such as rearrangements, copy number alterations, insertions, and deletions, at an acceptable cost [44]. NGS techniques are high throughput and can analyse multiple DNA sequences in parallel. Their multigene panels represent a targeted approach to sequence several genes simultaneously [45]. These panels can be individualised according to tumour types and differ according to the genes being analysed. For example, sinonasal tumour-specific panels have been proposed that can analyse numerous susceptibility genes for this tumour type [46]. A multigene panel can be tailored to genes of interest and can be updated as new genes are discovered [45]. These genomic advances not only pave the way for the development of targeted therapies for different molecular subclasses of tumours but also allow for more precise diagnoses based on molecular profiling and delineate subgroups of patients who are more likely to benefit from targeted agents [47,48]. These tests can also identify actionable driver mutations and underlying mechanisms of drug resistance to reveal patients who are likely to be resistant to therapy [47]. The number of FDA-approved companion molecular diagnostic assays is growing. The approval of these NGS panels suggests that NGS testing is becoming a standard of care in oncology, and the paradigm is shifting from single-gene assays to an NGS approach that can detect the patient’s entire mutational catalogue [49]. Importantly, any molecular result must be always interpreted with the clinical context of the patient and correlated with the pathological findings.

Approaches to biopsies are also evolving. Liquid biopsies as a biomarker for disease detection, prognostication, and therapeutic intervention in head and neck cancer and cancer in general have been developed to detect molecular alterations when tissue biopsies are not feasible [50]. Liquid biopsies detect alterations in circulating tumour DNA (ctDNA), which are small fragments of DNA released by malignant lesions [51]. They hold great potential as a non-invasive, rapid, repeatable adjuvant tool for cancer detection and surveillance. However, there are still ongoing challenges—including limited specificity, sensitivity, and lack of standardization—although their potential applications are rapidly expanding.

### 2.3. Ancillary Techniques

The recently described novel methods could help in establishing the pathological diagnosis of tumours. However, their accuracy and reliability are still unclear, and further studies need to be carried out to validate them.

Ultrasound elastography (USE) is currently under investigation, and it describes a variety of ultrasound-based imaging techniques that measure tissue stiffness properties. Most of the published data belong to pilot studies. Most studies have documented promising results in terms of high accuracy for malignancy in thyroid nodules [52] and cervical lymph nodes [53]. However, other studies have documented opposite results [54]. USE appears to be suboptimal for salivary neoplasms [55], and some evidence suggests that USE does not provide additional diagnostic information, especially in small volume lesions. The role of this technique in routine head and neck assessment is currently unclear, although preliminary evidence provides ample justification for further research in this area.

Raman spectroscopy may rapidly and accurately discriminate between healthy and malignant tissue [56,57]. This technique has hardly been used and is at an early stage. Preliminary analyses of the Raman spectra indicate that discrimination between diseased and healthy tissue is possible [57].

## 3. Quantitative Diagnosis

The quantitative diagnosis is the identification of a mass lesion and estimation of the tumour volume, which is mainly provided by the radiologist using imaging techniques. Unlike a pathologist, the radiologist provides a quantitative diagnosis and may suggest a specific differential diagnosis. For instance, the radiologist is often able to establish whether the lesion is cystic, infiltrative, or vascular but cannot give a histological diagnosis.

Advances in diagnostic imaging have been continuous in recent years with the introduction of new technologies. There are multiple modalities of imaging with corresponding functions in diagnosis, staging, and management of HNC. Ultrasound, computed tomography (CT), magnetic resonance imaging (MRI), and nuclear medicine techniques (e.g., positron emission tomography (PET)) are the most used imaging modalities. All these modalities of imaging have a strong influence on treatment planning, and therefore, an understanding of the merits of each type and timing of optimal imaging for anatomical characterization and accurate staging is essential.

### 3.1. Ultrasound

Ultrasound is the technique of choice for the initial approach to a cervical mass and could provide clinical decision support. It allows for assessment of the size, location, composition, and relationship to the great vessels to be determined. It is easily accessible and can provide excellent, non-invasive soft tissue characterization when assessing superficial primary sites and nodal basins. In a recently published meta-analysis by Huang et al. [58], it was observed that the sensitivity, specificity, positive likelihood ratio (LR), negative LR, and diagnostic odds ratio of ultrasound for diagnosing lymphadenopathy were 0.88 (0.86–0.90), 0.90 (0.88–0.92), 6.04 (3.67–9.95), 0.15 (0.10–0.21), and 47.38 (23.45–95.66), respectively. Some authors have reported that the sensitivity of ultrasound for the detection of cervical lymph node metastasis may be superior to that of CT (69 vs. 45%) [59], and for routine diagnosis of nodal spread in the neck, ultrasound could be recommended [60].

For salivary gland lesions, thyroid nodules, and lymph nodes, ultrasound provides excellent image resolution, and it is the preferred modality of imaging for guided diagnostic interventional procedures, such as FNAC and core needle biopsy. The use of intraoral ultrasound to analyse the depth of invasion in oral cavity SCC is currently expanding [61]. The limitations of ultrasound are its reliance on operator skills and inability to assess deeper structures due to limited soft tissue penetration and overlying bone or air artefact.

### 3.2. Computed Tomography (CT)

CT is widely used in oncology and can be applied to assess primary site of HNC, nodal disease, and staging as a part of the established TNM staging system. The use of high-resolution image acquisition with thin slices allows for good-quality multiplanar reconstructed images for anatomical demonstration and treatment planning. The slice thickness of multidetector CT depends on the type of scanner, although a collimation of 1–1.5 mm is generally used. Multiplanar images are reformatted to a thickness of 2–3 mm, and in some situations, high-resolution sections as small as 0.625 mm can be acquired depending on the scanner’s capability. Iodinated contrast can be applied to highlight blood vessels to enhance contrast between soft tissue structures and to assess the calibre and patency of carotid vessels along with evaluating the nature of highly vascular tumours. Without contrast, it can be difficult to depict abnormal soft tissue and detect pathological lymph nodes, particularly throughout the neck and those within proximity to vascular structures.

Sensitivity of CT is around 70% in primary tumours and 65% in recurrent carcinomas. Specificity is 70% in primary tumours and 80% in recurrent neoplasms. When assessing neck nodes, sensitivity and specificity are higher (around 85% and 95%, respectively). In recurrent lymph node metastases, sensitivity is 70% and specificity 90% [60,62].

Disadvantages of CT compared to other imaging modalities include radiation dose burden, risk of contrast-induced nephropathy and allergic reaction, and artefacts secondary to dental amalgam/orthopaedic prostheses. The mean radiation dose of a CT neck with IV contrast is 3.9 mSv and for CT Thorax is 8 mSv [63].

When compared to MRI, CT provides an excellent assessment of the infrahyoid neck, minimising any motion artefacts due to its fast acquisition time. It can provide a better assessment of the bony involvement and depiction of calcifications. At the same time, it allows simultaneous assessment of the thorax for paratracheal and upper mediastinal lymph nodes, pulmonary metastases, or synchronous primary lung lesions.

Recent data from studies with new-generation dual-energy CT systems suggest an improved detection of tumour invasion into cartilage, cortical bone, and bone marrow [64,65].

### 3.3. Magnetic Resonance Imaging (MRI)

MRI is the preferred modality for local assessment of suprahyoid neck, including nasopharynx, sinuses, oral cavity, and oropharynx. It provides superior soft tissue contrast compared to other modalities, becoming suitable for assessing deep infiltration of the primary tumour and separation of oedema from tumour infiltration. It can also provide assessment of bone marrow invasion and regional nodal disease.

In general, sensitivity is similar or slightly higher than that observed with CT (80%), while specificity is higher in most studies (<90%) [62].

While T1-weighted sequences provide excellent anatomical assessment, T2-weighted sequences are particularly of value in characterizing soft tissue and further differentiating post-treatment fibrosis from residual tumour. Gadolinium sequences are of value in detecting subtle or occult early disease, which may not be visible on other modalities due to the dynamic vascular nature of malignant processes. They are also used to accurately define tumour margins, particularly with respect to skull base and perineural extension. Diffusion-weighted imaging (DWI) is useful in defining treatment response. DWI provides a quantitative measure (apparent diffusion coefficient—ADC) of restriction of free diffusion of water molecules in tissue, which is related to the microstructure of the tissue (e.g., cellular density). Hypercellular tumours and pathological lymph nodes show diffusion restriction and hence low ADC values, while oedema, inflammatory, post-therapeutic changes, and necrotic processes present low cellularity and high ADC values [66]. ADC values seem to have higher diagnostic accuracy over anatomical MRI for the primary tumour location, and they are relevant for response evaluation of treated head and neck tumour patients [67]. In DWI, it is also possible to model the Intravoxel incoherent motion (IVIM), which is biomarker for tissue microcirculation. IVIM biomarkers can be used for tumour characterization, especially in salivary gland tumours, and preliminary data show that (early) changes in IVIM parameters have predictive and prognostic value [68].

Finally, dynamic contrast-enhanced MRI perfusion imaging can be of value in detecting and quantifying the tissue microvascularity, providing characterization for HNC. This technique may help in assessing the histological grade of some tumours [69] and can improve detection of unknown primary tumours in addition to DWI and ^18^FDG-PET/CT in patients presenting with cervical SCC lymph node metastasis [70]. Similarly, MRI spectroscopy can be applied, providing a molecular breakdown of biochemical composition and evaluating the presence of specific metabolites. Therefore, it has the potential to differentiate between non-malignant tissue, malignancy, and post-radiation changes [71]. MRI is subjected to motion of artefacts resulting from breathing or swallowing, and it has some limitations for use in patients with renal impairment, metal prostheses and implants, and pacemakers.

### 3.4. Positron Emission Tomography (PET)

PET is combined with whole body CT for anatomical detail, using the radiolabelled 18 fluorodeoxyglucose (^18^FDG) tracer to demonstrate areas of increased metabolic activity. FDG concentration and distribution can be directly measured and correlated with primary or metastatic disease. This would help to differentiate malignant tissue from post-treatment changes, scarring, and benign lesions. ^18^FDG-PET-CT is currently indicated as a part of staging for SCC if there is an evidence of cancer spread beyond the primary site, mainly in advanced stages (III and IV) [3]. Moreover, in patients who have undergone chemo-radiotherapy (CRT), PET-CT is the most sensitive modality for assessing persistence or recurrences when performed at three months following CRT. After treatment, ^18^FDG PET/CT show better sensitivity than clinical examination or conventional imaging (CT and/or MRI) in terms of local, regional, and distant recurrence. Additionally, it can be used to identify an unknown primary in the presence of neck lymphadenopathy and no obvious primary site on MRI or CT. Although PET-CT imaging has a high negative-predictive value [72], it can have false positives, as in the case of the inflammatory processes. The positive-predictive value is reported in the order of 50% [73]. Moreover, it may not detect lesions smaller than 1 mm, and the rate of false negative may not be negligible [74]. New PET radiotracers have been developed and used in HNC. For instance, ^68^Ga-NODAGA-RGD has been used for tumour angiogenesis characterization [75].

## 4. Artificial Intelligence

There has been an exponential growth in the application of artificial intelligence (AI) in radiology and in pathology. This is resulting in the innovation of deep-learning (DL) and machine-learning (ML) technologies that are specifically aimed at cellular and radiological imaging and practical applications that could transform diagnostic pathology and radiology [76,77]. DL and ML have become the focus of research in the field of IA, despite its lack of explainability and interpretability. DL mainly involves automated feature extraction that can classify radiological and pathological images.

Advances in the AI field have enabled to improve consistency in cancer diagnosis and stratification. ML is a branch of AI that uses computational methods to detect patterns, gather insight, and make predictions about new data by using historical information that has been learnt. As the volume of training data increases, ML algorithms can produce more accurate and efficient predictions. DL is a subfield of ML in which algorithms are structured to create artificial neural networks with multiple hidden layers. All these methods have gained significant popularity in recent years because of their relatively high diagnostic accuracy.

Conventional tumour diagnosis is based on histopathological evaluation by light microscopy of tissue sections from biopsies or surgical resections in addition to clinical and radiological testing. These approaches can be time consuming and are prone to observational errors or variations in interpretation, which can lead to inconsistencies in cancer classification and prognosis [78,79]. Consequently, this can cause inaccuracies in diagnosis, which can have significant implications on patient management. Indeed, improvements in the accuracy of prediction could greatly assist healthcare professionals in early detection and planning optimal patient-specific treatments to reduce the burden of disease. However, a recent systematic review that analyses and describes the application and diagnostic accuracy of AI methods for detection and grading of pre-cancerous and cancerous head and neck lesions identifies a lack of evidence for these methods [80]. Authors highlight that the overall quality of evidence in studies is low, mainly due to the use of small, unicentric data sets and a high risk of bias that could have overestimated model accuracy rates.

Radiomics is a method that extracts large numbers of quantitative features from mostly cross-sectional medical images (CT, MRI, and PET) using data-characterization algorithms. After being trained using many exams and images, mathematical algorithms can analyse the medical images to identify patterns that provide information about abnormal findings mostly invisibly to the human eye. Differences in image intensity, shape, or texture can be quantified by radiomics analysis. AI and ML have shown the potential to use currently unused data available on patients’ medical imaging scans for building predictive models that lead to improved HNC characterization, prediction of treatment response, and survival [81]. Most experiments so far have been proof of concepts and conducted using a small number of samples acquired from a single or a few sites following a single protocol. However, successful deployment of ML-based models in a clinical setting will require algorithms based on large-scale studies that encompass various sites and variations, enabling the future ability to generalize.

Recent studies have reported potential applications of radiomics analysis for molecular classification, prognostic characterization, and treatment response prediction in patients with HNC [82,83]. Kann et al. [84] pointed out that DL could have utility in the identification of extranodal extension in patients with HNC and could therefore be integrated into clinical decision making. Haider et al. [85] concluded that radiomics imaging features extracted from pre-treatment PET/CT may provide complimentary information to the current AJCC staging scheme for survival prognostication and risk-stratification of HPV-associated oropharyngeal carcinoma. Mes et al. [86] reported that MRI radiomics not only can predict overall survival and relapse-free survival some tumours, but it also provides additional prognostic information to known clinical variables with the best performance of the combined models. Moreover, radiomics-based MRI phenotyping could differentiate oropharyngeal SCC according to HPV status, being a potential imaging biomarker [87]. Tandini-Lang et al. [88] found 25 studies in the radiomics literature that assessed its ability to predict the biological characteristics and phenotype of HNC and 28 studies that dealt with the prediction of post-treatment events. Only three of these 53 studies did not identify a statistically significant role for radiomics. All these studies, however, were retrospective, and none of the identified radiomic samples (signatures) were validated in an independent prospective cohort. Finally, another application of radiomics would be the delineation of the tumour volume in the planning of RT. A fully automatic tumour volume-contouring method based on PET-CT changes has been successfully proposed with high accuracy and efficiency by some authors [89,90].

Nevertheless, standardization of radiomic analysis pipelines, image acquisition protocols, and targets should be strengthened to pave the way for the daily clinical use of these techniques and, ultimately, for better outcomes and reduced treatment-related toxicities in the field of HNC.

## 5. Discussion

**Figure 1:**
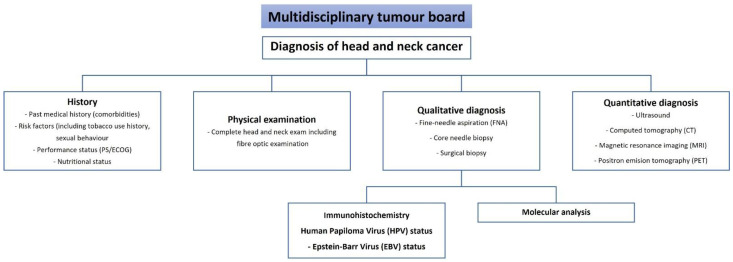
Flow chart for the head and neck cancer diagnosis.

The final diagnosis of HNC cancer is based on the clinical, radiological, and pathological assessment of the tumour (Figure 1). All these components complement the subjective assessments and limit the inaccuracy of the diagnosis. The art of diagnosis reflects the knowledge, competence, and practice of a multidisciplinary team of physicians. The roles of the pathologist and radiologist are complementary to that of the practicing clinician. Discrepancies between clinical, pathological, and radiological findings should be considered carefully and may indicate the need for further pathological, radiological, and clinical investigations.

To avoid patient mismanagement and unnecessary complications, it is mandatory to establish the correct diagnosis before initiating definitive treatment. The choice of treatment is an important parameter that influences both the prognosis and the patient’s QoL. Regardless of the diagnostic method used, our goal is to make a speedy and early accurate diagnosis, and only then can appropriate and adequate therapy be recommended to the patient.

A precise cancer diagnosis provides an accurate assessment of the tumour, including the cell type and stage, and helps physicians determine the most appropriate plan for treatment. The pathological diagnosis is the definitive diagnosis and should guide the proposed treatment. A multitude of techniques, many of them complementary, are currently available to establish an adequate pathological diagnosis. Moreover, the continuous advance of molecular medicine has made it possible to establish increasingly more specific diagnoses, allowing for the application of personalised treatments. However, caution must be exercised when applying the new technologies, as many of them are still in the validation phase.

Radiology is also essential in defining treatment. The aim of imaging in HNC is to establish the extent and size of the tumour and to distinguish the recurrent tumour from post-treatment changes. Diagnostic imaging techniques are becoming increasingly powerful and make it possible to detect small amounts of tumour and to define its precise location. The implementation of AI algorithms in diagnostic radiology is enabling radiologists to perform higher value-added tasks, being more visible to patients and playing a vital role in multidisciplinary clinical teams.

As shown above, the discrimination between qualitative and quantitative diagnosis is not always clear. However, these types of diagnosis can be of additional value. For example, in sentinel lymph node biopsy, presence of positive sentinel lymph nodes and size of tumour deposits within these sentinel lymph nodes, e.g., isolated tumour cells (size ≤ 0.2 mm), micrometastasis (size > 0.2 mm and ≤ 2 mm), and macrometastasis (size > 2 mm), may be used to predict survival and individualize treatment planning [91]. Another example is the use of the combination of lymph node size and (semi-quantitative) standardized uptake value (SUV) ^18^FDG to select lymph nodes for ultrasound-guided, fine-needle aspiration cytology [92]. Qualitatively and quantitatively analysed DWI and ^18^FDG-PET-CT are valuable for response evaluation after CRT [93]. Functional imaging parameters, performing DWI/Intravoxel incoherent motion (IVIM), Dynamic Contrast Enhancement (DCE-)MRI, and ^18^FDG-PET-CT yielded complementary value in capturing tumour characteristics and added to estimated locoregional and overall survival [94].

## 6. Conclusions

The pathologist plays a crucial role in the MTB. In nearly all cases, a tissue diagnosis is required to confirm the disease process before treatment begins. Even in settings where the diagnosis appears straightforward, a timely and appropriate report is necessary for the comprehensive discussion. Moreover, when malignancy is suspected, radiology must be performed to obtain precise anatomical details regarding the tumour localization and extension, which are critical in determining operability or in planning RT or the need of concomitant CT.

## Data Availability

No new data were created or analysed in this study. Data sharing is not applicable to this article.
